# Editorial: Molecular Characterization of Clinically Important Gram-Negative Bacteria Recovered From the Environment: Antimicrobial Resistance, Virulence and Epidemiology

**DOI:** 10.3389/fmicb.2022.962840

**Published:** 2022-07-08

**Authors:** Gerson Nakazato, Helia Magaly Bello-Toledo, Anyun Zhang, Eliana Guedes Stehling

**Affiliations:** ^1^Laboratory of Basic and Applied Bacteriology, Department of Microbiology, Biology Sciences Center, Londrina State University (UEL), Londrina, Brazil; ^2^Laboratorio de Investigación en Agentes Antibacterianos (LIAA), Departamento de Microbiología, Facultad de Ciencias Biológicas, Universidad de Concepción, Concepción, Chile; ^3^Animal Disease Prevention and Food Safety Key Laboratory of Sichuan Province, Key Laboratory of Bio-Resource and Eco-Environment of Ministry of Education, College of Life Sciences, Sichuan University, Chengdu, China; ^4^Departamento de Análises Clínicas, Toxicológicas e Bromatológicas, Faculdade de Ciências Farmacêuticas de Ribeirão Preto, Universidade de São Paulo (USP), Ribeirão Preto, Brazil

**Keywords:** molecular characterization, Gram-negative bacteria, multidrug resistance, antimicrobial resistance genes, horizontal gene transfer

The emergence of antimicrobial resistance (AMR) is due to different factors, including incorrect and excessive use of antimicrobials. The environment is increasingly being recognized as an important reservoir of AMR and plays a key role in the dissemination of antimicrobial resistance genes (ARGs). Thus, the spread of multidrug-resistant (MDR) bacteria and ARGs to diverse environmental compartments is of concern. Although the species of Gram-negative bacteria such as *Acinetobacter baumannii, Klebsiella pneumoniae, Escherichia coli*, and *Pseudomonas aeruginosa* are the most frequently reported infections with MDR phenotype, many other species in this group are also MDR. Therefore, characterization of clinically important bacteria recovered from the environment is one of the key checkpoints within the One Health concept.

In this context, seven articles were published in this Research Topic. All these articles indicated the importance of monitoring and molecular typing of clinically important AMR bacteria and the corresponding ARGs from different sources. Although several studies on AMR have been reported in the literature, new AMR phenotypic and genotypic data were presented in these articles.

Janssen et al. characterized a *K. pneumoniae* strain from an urban lake in Brazil obtained during a drug-degrading bacterial prospection. The strain belonged to a new sequence type, ST5236 KL45:O1v2, and carried ARGs encoding β-lactam, acriflavine and fosfomycin resistance, as well as the genes for metal tolerance. The *bla*_CTX−M−15_ gene was detected in proximity to the Siphoviridae genes, while the *bla*_KPC−2_ gene was found within a novel non-Tn*4401* genetic element. The latter was located on an extrachromosomal contig and was identical to a contig from a *K. pneumoniae* isolate from a nearby hospital. These findings indicate a putative gene flow from the hospital network into the Paranoá Lake, which is worrying since this lake is used for recreational purposes and as an environmental buffer for the reuse of drinking water.

Hao et al. characterized *K. pneumoniae* strains from infected pancreatic necrosis (IPN) samples. The authors used whole-genome sequencing (WGS) of eight strains to detect 42 ARGs on the chromosome and 27 on plasmids. The strains showed MDR phenotypes and were classified in three different types according to the AMR phenotypes for resistance to ceftazidime-avibactam (CZA) and aztreonam (ATM). The higher resistance to CZA was found in strains co-harboring *bla*_NDM−5_, *bla*_OXA−1_, *bla*_CTX−M−15_, and *bla*_SHV−187_, which presented higher resistance to ceftazidime than the *bla*_NDM−5_ gene, suggesting that the coexistence of extended-spectrum β-lactamases (ESBL) and metal-β-lactamase is currently a major challenge for antimicrobial therapy. In addition, strains co-harboring *bla*_CTX−M−65_, *bla*_SHV−182_, and *bla*_TEM−181_ were less resistant to β-lactams when compared to other ESBLs. Authors also showed that ATM plus avibactam inhibited *K. pneumoniae* more significantly and proposed this drug combination for therapy of complex infectious caused by MDR *K. pneumoniae* strains.

Zhang et al. investigated amikacin heteroresistance in *K. pneumoniae* strains and used WGS and quantitative reverse-transcription PCR (qRT-PCR) to explore the potential mechanism of amikacin heteroresistance. For this, 155 *K. pneumonia*e strains were analyzed and 13 of them (8.39%) were classified as amikacin-heteroresistant. The results revealed that the majority of the heterogeneous phenotypes (84.61%) were unstable and the frequency of heteroresistant subpopulations ranged from 2.94 × 10^−7^ to 5.59 × 10^−6^. WGS analysis showed nucleotide and amino acid polymorphism in various genes between the amikacin-resistant and amikacin-heteroresistant subpopulations. The qRT-PCR analysis showed that an increased expression of genes related to aminoglycoside resistance in amikacin-heteroresistant strains may be associated with amikacin heteroresistance, raising concerns regarding the emergence of amikacin-heteroresistant *K. pneumoniae* strains. Potentially, amikacin therapy of *K. pneumoniae* may select for high-level resistance due to the presence of heteroresistant subpopulation.

Tartor et al. investigated the prevalence and antimicrobial resistance profile of bacterial isolates from clinical and subclinical bovine mastitis and raw unpasteurized milk. The most prevalent MDR species were *K. pneumoniae, Enterobacter cloacae*, and *Aeromonas hydrophila*, followed by *E. coli* and *Proteus mirabilis*. An extensive drug resistance phenotype was also found in strains of *E. coli* and *P. mirabilis*. Ten strains (four *E. coli*, three *P. mirabilis*, and three *Klebsiella* sp.) were analyzed by WGS. In one *K. pneumoniae* strain from bovine milk, an IncFIB plasmid carried fosfomycin resistance gene, *fos*A5, and the colistin resistance gene, *mcr-10*, which was bracketed by xerC and insertion sequence IS*26*. This is the first report of *K. pneumoniae* co-harboring the *mcr-10* and *fos*A5 genes from bovine milk in the Middle East, reinforcing the importance of prudent use of antimicrobials in animals, as well as the need for AMR surveillance.

Zhang et al. characterized a novel AmpC, encoded by the *bla*_PRC−1_ gene, which was located on the chromosome of a *Pseudomonas wenzhouensis* strain isolated from the sewage discharge of animal farm in China. PRC-1 is a 379-amino acid AmpC β-lactamase sharing a 57.7% amino acid identity with the functionally characterized AmpC enzyme PDC-211 from *P. aeruginosa*. It confers resistance to β-lactams, such as penicillins and cephalosporins, and it is inhibited by avibactam. The genetic context of *bla*_PRC−1_ was evaluated and no mobile genetic element was predicted. Authors reinforce the importance of the identification and characterization of novel β-lactamase-encoding genes, showing that numerous unknown resistance mechanisms in environmental bacteria may pose potential risks to human health.

Díaz-Gavidia et al. analyzed surface water samples from 12 sampling sites in Maipo and Maule rivers central Chile from August 2017 to April 2019. In Maule river fecal coliform levels ranged between 1 and 130 most probable number (MPN) per 100 mL, while in Maipo river between 2 and 30,000 MPN/100-mL. AMR bacteria were detected in both rivers and 37.5% of *E. coli* isolates were MDR. In addition, 5.1 and 6.6% of other environmental strains from Maule and Maipo rivers, respectively, showed MDR phenotypes. The authors concluded that a major fecal contamination of the two rivers is due to agriculture and urban land uses. There is a need to decrease untreated discharges into these rivers to reduce the burden of water-borne diseases and AMR in the region.

Zhang et al. validated a rapid (60 min) AMR test using SYBR Green I and propidium iodide (PI) staining. The test targeted resistance to cefepime (CPM), ceftriaxone (CRO), meropenem (MPM), and ciprofloxacin (CIP) and was applied to 100 clinical strains of *K. pneumoniae*. The authors observed that the AST rapid test could detect antimicrobial resistance to all tested drugs in 60 min, corroborating the conventional methods of Kirby-Bauer and broth microdilution with an accuracy of 93, 96, 96, and 99 for CIP, CPM, MPM, and CRO, respectively. In this context, the authors concluded that the rapid AST test can help to reduce the empirical use of antimicrobial agents and, consequently, decrease the health and economic burden of AMR.

Articles in this topic demonstrated the importance and advantages of using genomics in the characterization of potentially pathogenic MDR strains, with a focus on ARGs and genotyping. In addition, a new approach can be used to quickly determine the AMR profile of bacterial strains. Therefore, this Research Topic demonstrated the dissemination of potentially pathogenic MDR strains into different ecological compartments, which may serve as AMR hotspots.

## Author Contributions

All authors listed have made a substantial, direct, and intellectual contribution to the work and approved it for publication.

## Funding

This work was supported in part by the Fundação de Amparo à Pesquisa do Estado de São Paulo—FAPESP (grant no. 2021/01655-7), the Conselho Nacional de Desenvolvimento Científico e Tecnológico (CNPq) (grant no. 308914/2019-8) to ES and (grant no. 305414/2021-6) to GN, and the Key R&D Program of Sichuan province, China (grant no. 2022ZDZX0017) to AZ.

## Conflict of Interest

The authors declare that the research was conducted in the absence of any commercial or financial relationships that could be construed as a potential conflict of interest.

## Publisher's Note

All claims expressed in this article are solely those of the authors and do not necessarily represent those of their affiliated organizations, or those of the publisher, the editors and the reviewers. Any product that may be evaluated in this article, or claim that may be made by its manufacturer, is not guaranteed or endorsed by the publisher.

